# Antigen-Presenting Cells and T Cells Interact in a Specific Area of the Intestinal Mucosa Defined by the Ccl25-Ccr9 Axis in Medaka

**DOI:** 10.3389/fimmu.2022.812899

**Published:** 2022-02-03

**Authors:** Narges Aghaallaei, Rashi Agarwal, Joergen Benjaminsen, Katharina Lust, Baubak Bajoghli, Joachim Wittbrodt, Carmen G. Feijoo

**Affiliations:** ^1^ Centre for Organismal Studies (COS), Heidelberg University, Heidelberg, Germany; ^2^ Directors’ Research Unit, European Molecular Biology Laboratory (EMBL), Heidelberg, Germany; ^3^ Departamento de Ciencias Biologicas, Facultad de Ciencias de la Vida, Universidad Andres Bello, Santiago, Chile

**Keywords:** T cells, mucosal immune response, antigen-presenting cell, teleost fish model, intestine

## Abstract

Organized intestinal mucosal immune response appears to be restricted to tetrapods. In teleost fish, there is no evidence for the existence of a particular intestinal region that facilitates the interaction of antigen-presenting cells (APCs) and T cells, such as secondary lymphoid organs. Indeed, despite their importance in the defense against pathogens, the location and manner of APC-T cell interaction within the fish gut is unknown. Here, using non-invasive live imaging of newly developed transgenic reporter lines, we addressed the spatial organization and behavior of APCs and T cells in the intestine of medaka fish both during homeostasis and inflammation. We report that Ccr9a^+^ T cells are recruited to a band in the lamina propria next to the muscularis mucosa in which Ccl25-expressing cells are present. Ccr9a^+^ T cells contact APCs for several minutes, in a process mediated by connexin 43. This type of interaction was observed in homeostasis and inflammation, with the interaction being longer and more frequent during inflammation. Thus, our results demonstrate that the mucosal immune response in the intestine of medaka is organized and endowed with a specific region with specialized microenvironment and function.

## Introduction

One key feature of the mammalian mucosal immune system is its high level of organization, which warrants a highly efficient immune response. Secondary lymph organs (SLO), as the Peyer’s patch (PP) in the gut, are the proof of that. The wide repertoire of T cells with unique T cell antigen receptors that each individual has, implies an extremely low possibility for the encounter of a given T cell and the APC loaded with the reactive antigen (1). Thus, to overcome the challenge, SLO provide a defined area in which T cells and antigen-loaded APC concentrate, thereby facilitating considerably the encounter of the complementary APC-T cell couple (2). To this end, PP, as the others SLO, possesses mechanisms for the recruitment of T cells towards itself (3).

In non-mammalian vertebrates, a simplified version of SLO denominated lymphoid aggregates have been observed along the intestine of urodele and anuran amphibians such as the salamander *Pleurodeles waltl* (4) and the frogs *Bufo marinus*, *Rana pipiens*, *Bufo americanus* and *Xenopus leavis* (5). On the contrary, no SLO or any kind of intestinal lymphoid organized structure has been detected in teleost fish species yet. Indeed, it is generally accepted that the teleost fish gut lymphoid tissue is diffuse, with innate and adaptive immune cells randomly distributed along the lamina propria in the mucosa (6–8). Recently, the existence of encapsulated and unencapsulated lymphoid aggregates in intestinal mucosa were described in the African lungfish, an extant representative of the closest ancestral lineage to all tetrapods (9). Of note, lungfish lymphoid aggregates lack germinal centers, indicating that they have a lower level of organization than that found in mammals (9). This result demonstrates that gut mucosa equipped with defined sites to favor encounter of a T cell with the respective antigen-loaded APC evolved before tetrapod and with a simplified micro-architecture. Even more exciting, it opens the possibility of the existence of some kind of organization that facilitates the encounter of a complementary APC-T cell couple in the intestinal mucosa of more ancestral fish such as teleost. At the present, there are no antecedents in these fish that can give lights of where and how the interaction between T cells and antigen-loaded APCs occur in the gut, not even about their individual *in vivo* behavior during homeostasis. The signaling pathway essential for T cells homing to the small intestine in mammals, the Ccl25-Ccr9 axis (10), are conserved in fish (11). Transcriptional analysis of both genes after oral vaccination in sea bass showed an increase in their mRNA level (12). Likewise, the increase in T cells present in the gut of zebrafish larvae during an inflammatory process is accompanied by an increase in the intestinal *ccl25* mRNA level (13), suggesting possible function conservation. On the other hand, the existence of organized lymphoid tissue in other mucosal organ, the gills, was reported a long time ago (14). This interbranchial lymphoid tissue (ILT) consists mainly of T cells, embedded in a meshwork of epithelial cells, with few B cells and Mhc class II^+^ cells (15, 16). In salmonid, ILT T cells are mainly intraepithelial CD3ϵ^+^ T lymphocytes (14) and the mRNA expression of chemokine *ccl19* was detected in this tissue (17), suggesting a secondary lymphoid organ function. In non-mucosal organs, organized lymphoid tissue with hypothetical equivalence to organs with proven immune response functions have been already described in fish. A lymphoepithelial compartment with anatomical and developmental similarities to the bursa of Fabricius was described in the cloacal region of Atlantic salmon (18). This organ contains antigen-presenting cells and intraepithelial lymphocytes, in addition to expressing *ccl19* mRNA. Likewise, aggregates of highly pigmented macrophage, denominated melanomacrophage centers, present in the kidney, spleen and liver of several fish species have been proposed as a primitive site for adaptive immune system activation because of sharing morphological characteristics with mammalian germinal center in addition to increase size and number when exposed to pathogens (19–21).

To explore *in vivo* teleost fish intestinal T cells and APCs behavior under homeostasis and in response to inflammation, we took advantage of the simplicity and speed with which transgenic reporter lines can be generated in the teleost fish medaka (*Oryzias latipes*). We used an already established, Tg(lck:GFP) (22), and a new cell-type-specific reporter lines, Tg(il12p40b:mTq2), to label T cells and activated APCs respectively, and addressed their selective migration to a specific site in the gut lamina propria where they interact with each other, as well as the molecular compounds that regulate this process. Our data reveal the existence of a particular site in the lamina propria, defined by the Ccl25-Ccr9 axis, where T cells and APCs interact both during homeostasis and inflammation

## Materials and Methods

### Fish Maintenance

Medaka (Oryzias latipes) stocks are maintained as closed stocks at the Centre for Organismal Studies at Heidelberg University. Fish husbandry and experiments were performed according to local animal welfare standards (Tierschutzgesetz 111, Abs. 1, Nr. 1, Haltungserlaubnis AZ35-9185.64 and AZ35-9185.64/BH KIT in accordance with the European Union animal welfare guidelines. The experiments were conducted under laboratory animal handling protocol of the animal bioethics committee of the Universidad Andres Bello, Chile (certificate number: 020/2016). In the Tg(il12p40b:mTq2) line a cyan fluorescent protein variant, mTurquoise2 (mTq2), is expressed under the control of the 1.6Kb upstream of the il-12p40b gene. For the Tg(ccl25b:H2B-RFP) line, the red fluorescent protein fused with the human H2B protein was designed to be expressed under the control of a 4.7 Kb region that include a 3.6Kb element upstream of the start codon of the ccl25b gene and a 1.1Kb element including the first exon, a fraction of the second exon, and the first intron. Tg(ccl25b:GFP) used the same promoter element to express the green fluorescent protein. Other transgenic reporters in this study were described previously: The Tg(lck:GFP) was used to monitor T cells (22). The Tg(cxcr3a:GFP) was used to monitor mononuclear phagocytic cells, including dendritic cells and macrophages (23). The Tg(ccr9a:mCherry) was used to monitor ccr9a-expressing cells (22).

### Feeding Protocol

In this study, we used a fishmeal-based diet for the control group as previously described (24). To trigger mucosal inflammation, 75% of fishmeal was replaced by soybean meal. Both diets were formulated to be iso-energetic, iso-nitrogenous and iso-lipidic. For feeding, max. 45 freshly hatched larvae were maintained in 80ml fish water in a 100ml beaker in the fish facility to have optimal temperature and light cycle conditions. The fish water was changed daily. Larvae were fed twice a day, with an interval of 6 hours for 7.5 days and the last half-day fish were starved to avoid contents in the gut. A significant mortality rate in fish that were fed with the soybean meal-containing diet was not observed when compared to the control group. Feeding experiment were repeated in at least three independent times.

### Time Lapse Analysis

Confocal images were collected using a Leica SPE and SP8 system and processed using the Leica application suite X (LASX) or the Imaris Bitplane software. For live imaging, larvae were anesthetized in 1x Tricaine, Sigma Aldrich (St. Louis, Missouri, USA) diluted in 1x ERM and mounted in glass-bottomed Petri dishes (MatTek Corporation, Ashland, MA) in 1,3% Low Melting Agarose Roth (Carl Roth GmbH + Co. KG, Karlsruhe, Germany). The specimens were oriented laterally, facing down, so that the intestine was close to the cover-slip at the bottom of the dish. Imaging was performed on a Leica SP8 with a 20x immersion objective. At least 4 larvae were analyzed in each condition and the experiment were repeated in at least three independent times.

### *In Vivo* APC-T Cell Interaction Quantification

Tg(il12p40b:mTq2) was crossed with Tg(lck:GFP) and larvae were anesthetized and imaged as described above. Time lapses were recorded of the most posterior ~ 500 μm of the intestine, for 1 hour with a 1 min temporal resolution. Cropped 3D volumes of individual APCs were prepared for counting interactions with GFP+ cells, as the former were mostly stationary throughout the time lapses. Some crops had to be discarded because intestinal contractions made it impossible to follow the same APC for the full length of the recording. The number and length of interactions were manually counted by scrolling through 3D volumes for every time point. All processing were performed using Fiji (https://imagej.net/Fiji). At least 4 larvae were analyzed in each condition and the experiment were repeated in at least three independent times.

### Cryostat Sections

For cryosectioning, samples were fixed in 4% paraformaldehyde in PBS containing 0.01% Tween at 4°C overnight and cryopreserved in 30% sucrose at 4°C. They were mounted in 1.5% low melting agarose in 30% sucrose, placing head at bottom to ensure correct orientation to obtain transverse sections of the intestine, agarose blocks were cut and kept in 30% sucrose in PBS at 4°C overnight. These blocks were embedded in OCT compound (Tissue-Tek, Sakura), cryofrozen in liquid nitrogen and kept in cryostat (Leica) until it reaches temperature equilibrium. Sections of 30 µm were made and collected on Superfrost Plus slides (Thermo scientific) dried at 4°C overnight which were mounted with 60% glycerol (Sigma Aldrich). At least 8 sections in 6 larvae were analyzed in each condition and the experiment were repeated in at least three independent times.

### Immunohistochemistry Staining

For immunohistochemistry staining, dissected guts were fixed in 4% paraformaldehyde in PBS containing 0.01% Tween at 4°C overnight and then cryopreserved in 30% sucrose at 4°C. Guts were embedded in the OCT compound (Tissue-Tek, Sakura) and sectioned with 30µm thickness on a cryostat (Leica). Immunohistochemistry staining was performed as described previously (24). Briefly, to detect Connexin 43, sections were incubated with Connexin 43 Antibody (Cell Signaling, #3512) at a 1:500 dilution in blocking solution overnight at 4°C. After washing, a-conjugated Alexa 549 anti-rabbit antibody (1:500) was applied for overnight at 4°C. At least 8 sections in 6 larvae were analyzed in each condition and the experiment were repeated in at least three independent times.

### *In Situ* Hybridization Analysis

Whole mount *in situ* hybridization was performed with digoxigenin labelled antisense probes as described previously (25). After staining, the samples were fixed in 4% paraformaldehyde and cryosections of 30 μm were obtained on a cryostat. Imaging was done using Zeiss Axio Imager. At least 8 sections in 6 larvae were analyzed in each condition and the experiment were repeated in at least three independent times.

### Sudan Black Staining

Dissected guts were fixed in 4% paraformaldehyde in PBS containing 0.01% Tween for 4 hours and then incubated in Sudan black staining (Sigma-Aldrich) for 20min and washed 6 times, each for 10min. Finally, dissected guts were washed with absolute ethanol for 1-2 hours to remove the background. Dissected guts then were mounted in 80% glycerol for imaging. At least 20 larvae were analyzed in each condition and the experiment were repeated in at least three independent times.

### Quantitative RT-PCR

Total RNA was extracted from a pool of 40 dissected guts for each group using the TRIzol reagent (Ambion) according to the manufacture instructions. 1µg of RNA was used to synthetize cDNA with the Superscript First-Strand Reverse Transcriptase kit (Invitrogen) and hexamer primer. qPCR was performed with the ABI 7300 Real-Time PCR system using the Maxim SYBR Green/ROX qPCR Master Mix (2X) (Fermentas, Waltham, MA, USA) following the manufacturer’s instructions. Briefly, a 15 µL reaction volume was used, containing 1µL of 2-fold diluted cDNA. The PCR was run with a 10 minutes activation and denaturation step at 95°C, followed by 40 cycles of 30 s at 95°C, 30 s at 57-60°C, and 30 s at 72°C. Reaction specificity was verified using melting curve analysis and the absence of primer dimmers. Standard curves were obtained for each pair of primers by plotting Ct values against the log10 of five different dilutions of a cDNA mix solution for all analyzed samples. Real-time PCR efficiency (E) was calculated from a standard curve according to the equation E = 10(-1/slope). Relative expression was calculated with the Pfaffl method (26). Primers for qPCR are listed in **Table S1**. The experiments were repeated at least three times independently.

### Correlative Light and Electron Microscopy

Medaka larvae (20 dph) of the Tg(ccl25b:GFP) line were euthanized using a 20x tricaine solution and immediately fixed in 4% PFA + 0.1% GA in 100mM PHEM buffer and incubated overnight at 4 ºC. The posterior part of the gastrointestinal tract was dissected out and high pressure frozen using a HPM 010 (Bal-Tec, Liechtenstein) in a 200 μm deep aluminium planchet (Engineering Office M. Wohlwend GmbH, Sennwald, Switzerland) with 20% Polyvinylpyrrolidon molecular weight 10.000 (Sigma-Aldrich) in 0.1M PHEM buffer was to fill up the carrier for freeze protection. Freeze substitution was performed at - 90°C for 24 h with 0.1% (wt/vol) uranyl acetate in acetone according to Kukulski et.al. 2011 in a AFS2 freeze substitution unit (Leica Microsystems) and embedded in HM20 (Polysciences, Warrington, PA, USA). 200 nm thick sections were cut with a ultramicrotome (Ultracut UC7; Leica Microsystems) and a diamond knife (Diatome, Switzerland) and picked up on copper finder grids (Plan, Wetzlar, Germany) with a formvar film and carbon-coating. Grids were placed in PBS and sandwiched between two cover slips and fluorescence images acquired on a Zeiss Observer.Z1 inverted microscope (Carl Zeiss Microscopy GmbH, Oberkochen, Germany). Sections were then post-contrasted with 2% uranyl acetate in aqueous solution and Reynolds lead citrate solution, before electron microscopy was performed with a JEOL JEM-1400 electron microscope (JEOL, Tokyo) operating at 80 kV and equipped with a 4K TemCam F416 (Tietz Video and Image Processing Systems GmBH, Gautig) CMOS camera. Correlation of light and electron micrographs based on morphological features were done using the ec-CLEM plug-in for Icy (http://icy.bioimageanalysis.org/plugin/ec-clem/).

### Statistical Analysis

All experiments were performed at least in triplicate. Statistical analysis was carried out using Prism 9.1 (GraphPad Software). After a Shapiro Wilk normality test, the data was treated as not parametric, and analyzed with Mann Whithney U test or Kruskal Wallis test. Statistical significance was determined with a p value of 0.05.

## Results

### Myeloid and T Cells Are Enriched in the Posterior Intestine

**Figure 1:**
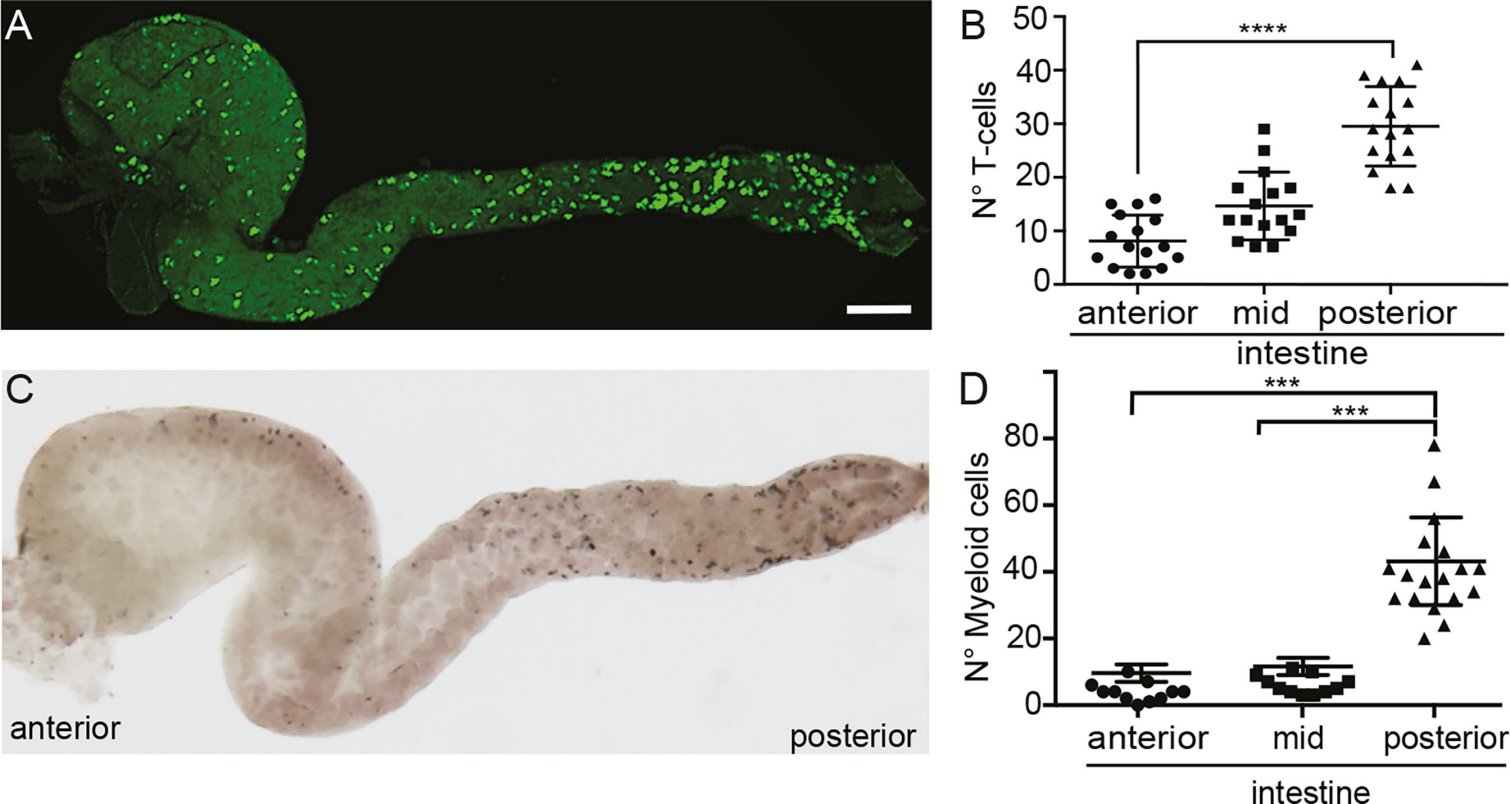
Localization of T cells and myeloid cells in the gut of medaka. **(A)** Representative image of a dissected intestine, including anterior, mid and posterior segments, of a Tg(lck:GFP) fish showing the distribution of GFP+ T cells. **(B)** Quantification of the number of GFP+ T cells in the three segments of the intestine. **(C)** Representative image of a dissected intestine of wild-type larva stained with Sudan-Black revealing the distribution of myeloid cells along the anterior-posterior axis. **(D)** Quantification of Sudan-Black+ cells in the anterior, mid and posterior gut. Statistical analysis was performed with Kruskal Wallis test. ***p < 0.001, ****p < 0.0001. Scale bars 100 µm. All assays were performed at least three independent times.

As teleost fish were assumed to lack organized lymphoid structures in the gut, the recruitment of T cells to a defined site to promote a coordinated interaction of T cells and APCs is not apparent. We first investigated the distribution of T cells along the intestine of medaka fish, to determine if they are stochastically dispersed or confined to specific sites. To this end, we used the Tg(lck:GFP), in which the green fluorescent protein (GFP) is expressed under the control of the *lck* proximal promoter, thus labelling T cells and allowing us to monitor their behavior in the gut *in vivo* (22). Dissecting the gut of these transgenic fish revealed an enrichment of T cells in the posterior segment compared to the anterior part (**Figures 1A, B**), similar to that observed for myeloid cells (**Figures 1C, D**), pointing to a higher presence of different components of the immune system in the posterior medaka intestine. This observation is comparable to the finding in carp (27) and rainbow trout fish (28).

### APCs and T Cells Respond to an Intestinal Inflammatory Stimulus

**Figure 2:**
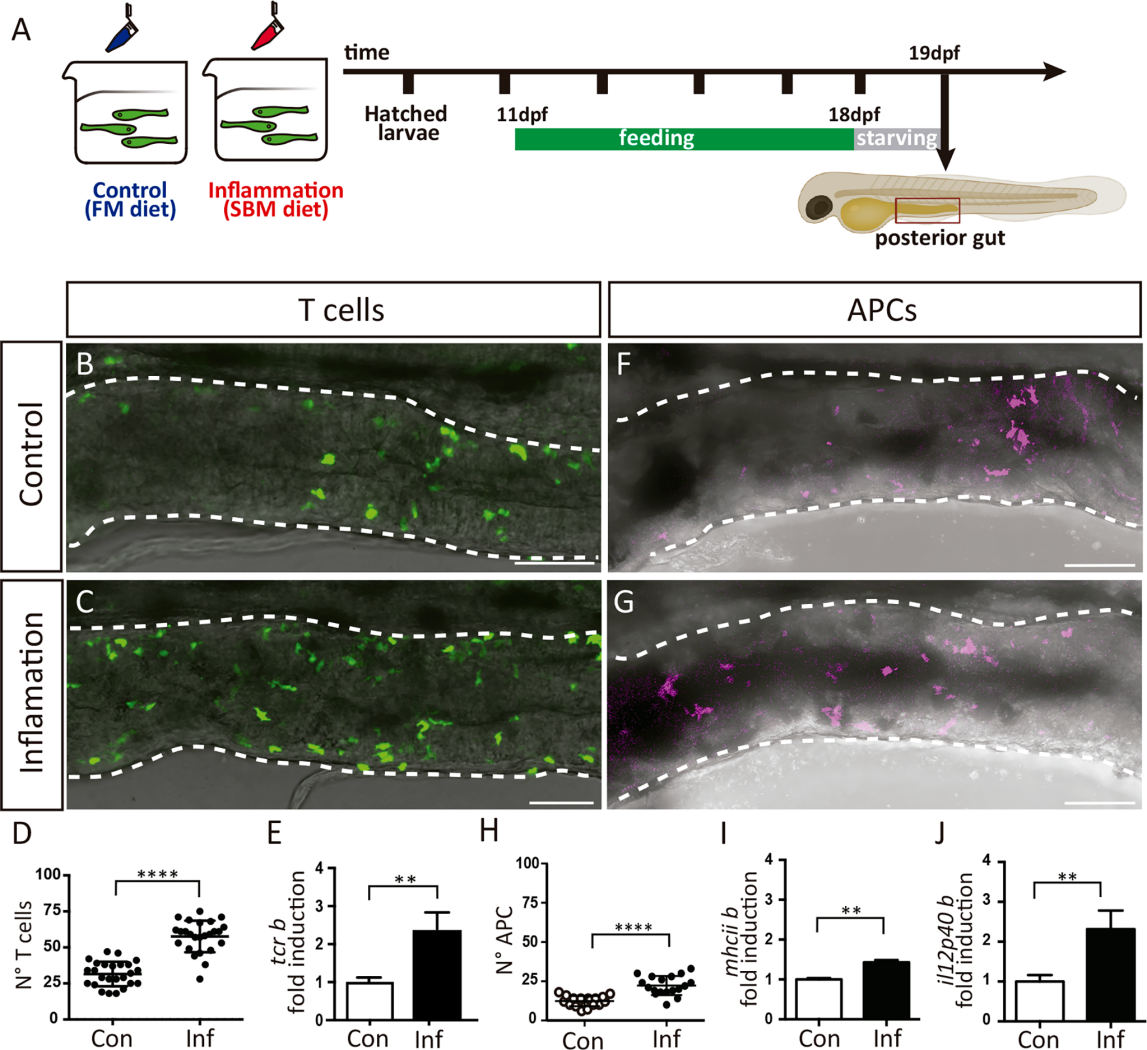
T cells and APCs response during intestinal inflammation. **(A)** Experimental strategy. Larvae were fed 8 days either with a control diet (FM) to maintain homeostasis, or with an inflammatory diet (SBM) to trigger inflammation (Inf). After a period of 12h of starvation, larvae were processed according to the different analysis. **(B, C)** Representative images showing the posterior intestine of a Tg(lck:GFP) fish in control and inflammatory condition. T cells are recognized by GFP expression. **(D)** Quantification of the number of GFP+ T cells in the posterior intestine in control and inflammatory condition. **(E)** Fold of mRNA induction of *tcrb* in the inflammatory condition respect to the control condition. The data was normalized against *elongation factor 1-alpha* (*ef1a*) gene. **(F, G)** Representative images showing the posterior intestine of a Tg(il12p40b:mTq2) fish in control and inflammatory condition. Antigen presenting cells (APC) are recognized by mTq2 expression. **(H)** Quantification of the number of mTq2+ APC in the posterior intestine in control and inflammatory condition. **(I, J)** Fold of mRNA induction of *il12p40b* and *mhciib* in the inflammatory condition respect to the control condition. The data was normalized against *elongation factor 1-alpha* (*ef1a*) gene. Statistical analysis was performed with the Mann-Whitney U test. **p < 0.01 and ****p < 0.0001. Scale bars 50µm. All assays were performed at least three independent times.

To activate immune response and thus stimulate APC-T cell interactions, we triggered intestinal inflammation in medaka fish using a soybean meal-based diet. It was previously shown that this diet induces an inflammatory response evidenced by the increase of neutrophil recruitment to the gut and the upregulation of pro-inflammatory cytokines such as *il-1* and *tnf1α* in zebrafish, common carp, and salmonid fish (24, 29–31). Medaka fish were fed either with the soybean meal-based diet (here after inflammatory diet) or standard fishmeal diet (here after control diet) for eight days before the analysis (**Figure 2A**). To confirm the induction of the intestinal inflammatory process, we addressed the activation of innate components of the immune system and found that the number of neutrophils and mononuclear phagocytes were significantly increased in the posterior intestine in inflamed (Inf) larvae compared to the control group (Con) (**Supplementary Figure 1**). Next, we analyzed if T cells respond to inflammation by increasing their presence in the posterior gut in Tg(lck:GFP) larvae, and indeed we observed an augment from an average of 32,08 cells in the control situation to 57,60 in inflamed larvae (**Figures 2B–D**). Congruently, the mRNA levels of the beta chain of the T cell receptor, *tcrb* were also significantly increased, with 2,34 times higher expression in the inflammatory condition compared with the control situation (**Figure 2E**). Finally, we also analyzed APC responses in the inflammatory condition and compared it to the control situation. To this end, we established a transgenic reporter line, Tg(il12p40b:mTq2), in which a segment of the Il-12p40b promoter drive the expression of the fluorescent protein mTurquoise2 (mTq2). Il12p40b is one of two different subunit that form IL-12, which is expressed by activated APCs in mammals and medaka (23, 32, 33), consequently mTq2 is expressed by these cells. We observed APCs in the gut in both conditions, with an increased presence in the inflammatory situation (12,39 v/s 22,13) (**Figures 2F–H**). Likewise, transcriptional levels of the beta chain of the major histocompatibility complex class II (*mhciib*) and *il-12p40b*, were upregulated in the gut of inflamed fish, been expressed 1,43 (p=0.02) and 2,31 (p=0.0002) times higher than in the control condition (**Figures 2I, J**), supporting the increase in APCs detected. Altogether, these results indicate that during the inflammatory process triggered, T cells and APCs respond to the inflammatory stimulus, and increase their presence in the intestine of medaka.

### APCs and T Cells Interact in the Lamina Propria During Homeostasis and Inflammation

**Figure 3:**
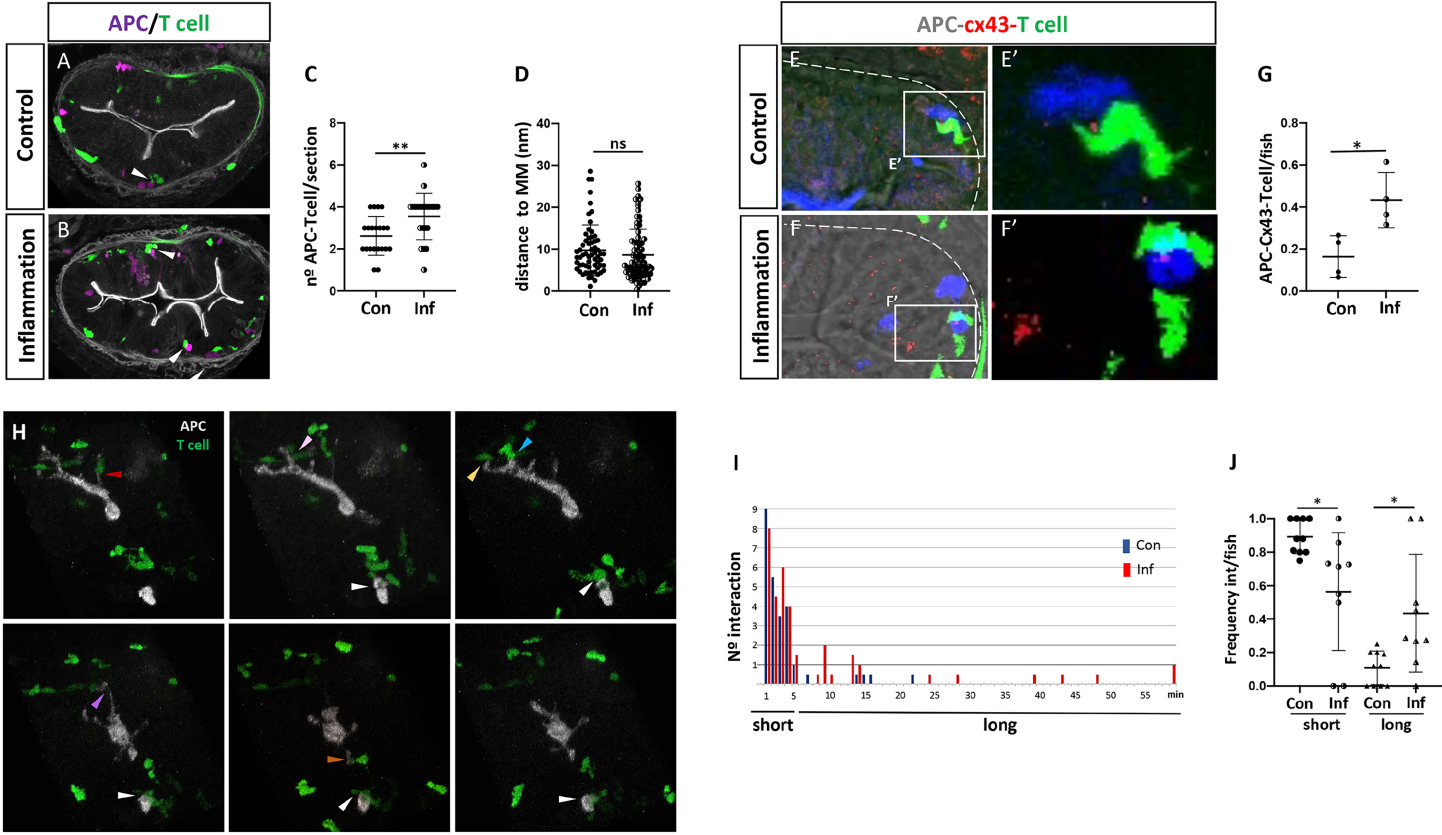
Behavior of T cells and APC cells in the posterior intestine in control and inflamed condition. **(A, B)** Representative images of a cryostat section of the posterior intestine of double transgenic Tg(il12p40b:mTq2;lck:GFP) fish showing the location of mTq+ APC (pink) and GFP+ T cells (green) in control and inflammatory condition. Arrowheads indicate APCs and T cells next to each other forming a couple. **(C)** Quantification of the number of APC-T cell couples per section in control and inflammatory condition. **(D)** Quantification of the distance of each couple to the muscularis mucosa per section in control and inflammatory condition. **(E, E’, F, F’)** Immunofluorescence to detect Cx43 performed in double Tg(lck:GFP; il12p40b:mTq2) fish in control and inflammatory condition. APC and T cells are shown in blue and green, respectively. **(G)** Quantification of the frequency of APC-Cx43-Tcell presence in control and inflammatory condition. **(H)** Images from a representative time lapse performed in a double transgenic Tg(il12p40b:mTq2;lck:GFP) fish highlighting the existence of short and long interactions. Arrow in white show an example of long APC-T cell interaction; arrows in different colors points examples of short APC-Tcell interactions Time is indicated in minute. **(I)** Histogram showing the number of short APC-T cell interaction (1-5 min), or long APC-T cell interaction (6-60 min), observed in the time lapse performed in control (blue) and inflammatory (red) condition. **(J)** Quantification of the frequency of short and long APC-T cell interactions observed in the control and inflammatory condition. Statistical analysis for **(C, D, G)** was performed with the Mann-Whitney test and for **(J)** with the Kruskal Wallis test. n.s., non-significant, *p < 0.05, **p < 0.01. All assays were performed at least three independent times.

Until now, there are no reports in any fish species demonstrating the interaction between APCs and T cells *in vivo* in any tissue or organ, neither in the presence of innocuous nor harmful exogenous antigens. With the two transgenic lines previously described, we generated a double transgenic fish [Tg(il12p40b:mTq2;lck:GFP)], in which APCs and T cells are fluorescently labeled in the same fish. Taking into account that in mammals the interaction between APCs and T cells in the intestinal lamina propria is a central step to the establishment of a tolerogenic or inflammatory response (34–36), we investigated if this interaction also occurred in this tissue in fish. First, we aimed to find APCs close to T cells and quantified the number of couples found in the gut of control and inflamed fish, analyzing cryostat sections of guts dissected from double transgenic fish. We found less T cell-APC couples in both control and inflamed gut, however the number of couples found in inflamed conditions was increased (control: 2.6 per section, inflamed: 3.6 per section) (**Figures 3A–C**). Independently of the condition, T cell-APC couples were always observed in a strip located at the same distance from the muscularis mucosa; 2,9-17,7 μm in control gut and 2,3-14 μm in inflamed gut (**Figure 3D**). To establish if the proximity between APCs and T cells was due to a cellular interaction was taking place, we evaluated the presence of the gap junction protein Connexin 43 (Cx43), a key component in the immunological synapse between T cell and MHC-peptide-loaded APC (37). We found that Cx43 was present at the contact site in T cell-APC couples in the control and inflammatory condition (**Figures 3E, E’, F, F’**), but in a higher frequency in the latter, 0,16 v/s 0,43 respectively (**Figure 3G**). Next, we evaluated T cells and APCs behavior *in vivo*, by performing one-hour time-lapse analysis. In both control and inflamed gut, we observed that most of the time T-cells migrated towards APCs, touched them and stayed in contact only for short periods of up to 5 minutes (**Figure 3H** color arrows, i; **Suplementary Movie 1**). Only on few occasions we observed prolonged interactions, of more than 5 minutes (**Figure 3H** white arrow, i; **Suplementary Movie 1**). In the inflammatory condition even some of them lasted the whole period of time analyzed, 60 minutes (**Figure 3I**). These two types of interactions occurred with different frequencies in the control and inflammatory condition. Short interactions were more frequent in control fish, and long interactions were significantly higher in inflamed fish (**Figure 3J**). The frequency of long interactions in the control and inflammatory conditions was similar to the APC-Cx43-Tcell interaction frequency observed in the corresponding condition; 0,16 and 0,11 in the control condition and 0,43 and 0,44 in the inflamed condition (**Figures 3G, J**). Taken together, these findings indicate that an interaction between APC and T cell lasts at least 6 minutes and take place in a specific region in the intestinal lamina propria of medaka fish.

### The Ccr9-Ccl25 Axis Regulate T Cells Recruitment to the Intestinal Mucosa, Defining a Specific Area for T Cell-APC Interaction

**Figure 4:**
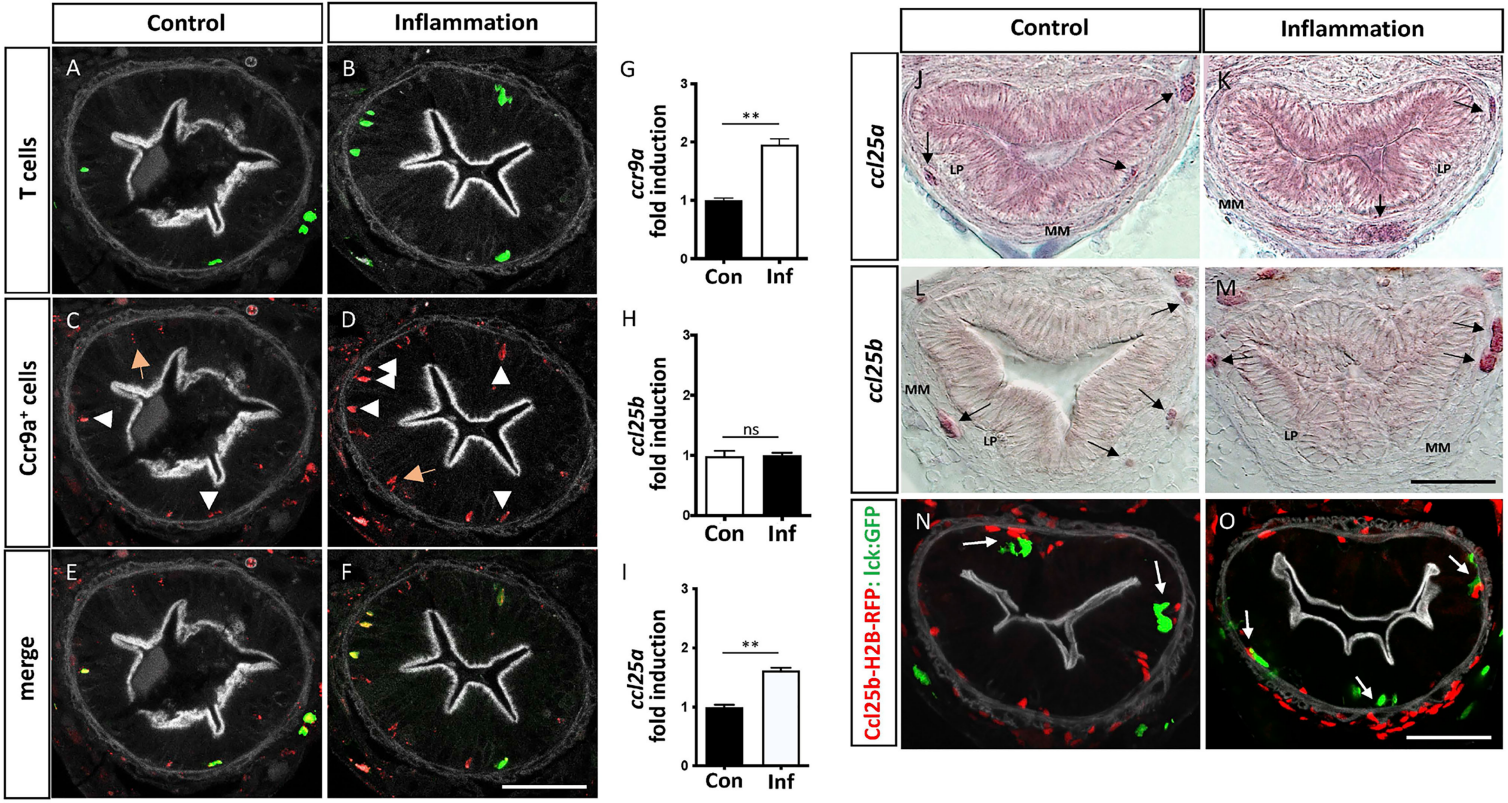
Intestinal T cells are Ccr9a+ and its ligand Ccl25a is expressed by mesenchymal cells. **(A–F)** Representative images of cryostat sections of the posterior intestine of double transgenic Tg(ccr9a:mCherry;lck:GFP) fish showing GFP+ T cells, Ccr9a-mCherry+ cells and their colocalization. Note, that all GFP+ T cells are also Ccr9a-mCherry+ (arrowhead) and a few Ccr9a-mCherry + cells are GFP- (orange arrow). **(G–I)** Fold of mRNA induction of *ccr9a; ccl25a* and *ccl25b* in the inflammatory condition respect to the control condition. The data was normalized against *elongation factor 1-alpha* (*ef1a*) gene. **(J–M)** Cryostat sections of the posterior intestine of wild type fish showing *ccl25a* and *ccl25b* mRNA expression pattern in the control and inflammatory condition, determined by *in situ* hybridization with a gene-specific riboprobes White arrows point mesenchymal cells expressing the corresponding mRNA. **(N, O)** Representative images of cryostat sections of the posterior intestine of double transgenic Tg(ccl25b:H2B-RFP; lck:GFP) fish, showing the location of Ccl25b-RFP+ cells (red) and GFP+ T cells (green) in control and inflammatory conditions. Statistical analysis was performed with the Mann-Whitney U test. n.s., non-significant, **p < 0.01. Scale bars 30µm. All assays were performed at least three independent times.

To further characterize APC-T cells interactions in the medaka gut, we aimed to address which molecular components regulate the recruitment of T cells to the intestinal mucosa. We focused on the CC-chemokine ligand 25 (Ccl25) and its sole chemokine receptor Ccr9 because of their key role in T cells recruitment to the gut in mammals (38). In medaka, the *ccr9* gene is duplicated, and both Ccr9a and Ccr9b are highly similar to mammalian CCR9 (11, 39). Taking advantage of the Tg(ccr9a:mCherry) transgenic line, in which all the Ccr9a-expressing cells are labeled (39), we examined the presence of this receptor in those T cells recruited to control and inflamed gut by analyzing the colocalization of mCherry and GFP in double transgenic fish Tg(ccr9a:mCherry;lck:GFP). We found that all the Lck-GFP^+^ cells were also *ccr9a*-mCherry^+^ (**Figures 4A–F**, arrowhead), indicating that the T cells present in this region express Ccr9a in both conditions. Also, some Ccr9a-mCherry^+^ were Lck-GFP^-^ (**Figures 4A–F**; arrow) denoting other cells than T-cells expressed Ccr9a in the gut. Of note, the transcriptional level of Ccr9a increased 2 times during inflammation compared to the control situation (**Figure 4G**). On the other hand, the medaka genome contains two paralogues of *ccl25, ccl25a* and *ccl25b* (39). No information regarding their expression in the gut is reported so far. Thus, we determined both mRNA expression pattern in control and inflamed gut and found that *ccl25a* and *ccl25b* have a similar pattern. In the case of *ccl25a*, it is expressed in the epithelium in homeostasis and inflammation. Also, the two chemokines are expressed in mesenchymal cells, either isolated or in clusters, present in the lamina propria and muscularis mucosa during (**Figures 4J–M**; arrow). We confirmed the mesenchymal identity of the Ccl25b^+^ cells performing correlative light and electron microscopy from the gut of Tg(ccl25b:GFP) larvae, in which the ccl25b proximal promotor drives the expression of GFP (**Supplementary Figure 2**). Transcriptional analysis supports these results, showing both chemokines are expressed in the gut and revealing *ccl25a* was upregulated in response to inflammation (**Figures 4H, I**).

To further explore the participation of the Ccl25-Ccr9 axis in the recruitment of T cells to the gut, we analyzed their location with respect to Ccl25^+^ mesenchymal cells in control and inflammatory condition. To this end, we used Tg(ccl25b:H2B-RFP) fish, in which the ccl25b proximal promotor drives the expression of a nuclear localized RFP, and crossed it with Tg(lck:GFP) fish to generate double transgenic Tg(ccl25b:H2B-RFP; lck:GFP) fish. Noteworthy, in control fish the few T cells present in the lamina propria were next to Ccl25b-RFP^+^ cells located in the muscularis mucosa, similarly to what was observed in inflamed fish. The only difference was that the number of T-cells present in this condition was higher (**Figures 4N, O**). Collectively, the obtained results strongly suggest that the Ccl25-Ccr9 axis control T cells homing to the intestine, defining a specific area for T cell-APC interaction.

**Figure 5:**
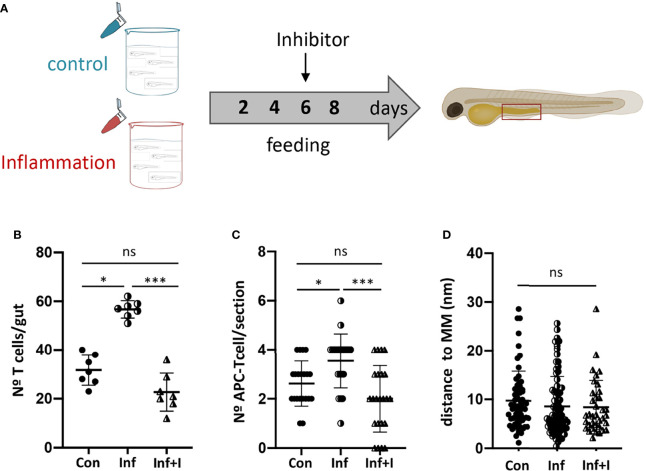
Ccr9-Ccl25 control APC-T cell interaction in the posterior intestine. **(A)** Experimental strategy used to inhibit Ccr9 receptor by treating fish with the specific inhibitor CCX282-B. **(B)** Quantification of the number of GFP+ T cells in the posterior intestine of Tg(lck:GFP) fish in control, inflammatory condition and treated condition. **(C)** Quantification of the number of APC-T cell couples per cryostat section in control condition, inflammatory and treated condition. **(D)** Quantification of the distance of each couple to the muscularis mucosa per section in control, inflammatory and treated condition. Statistical analysis was performed with the Kruskal Wallis test. n.s., non-significant, *p < 0.05, ***p < 0.001. All assays were performed at least three independent times.

To confirm this hypothesis, we employed a well-known Ccr9 antagonist, CCX282-B (40), to treat control and inflamed Tg(lck:GFP) fish and analyzed their impact on the number of T cells recruited to the gut (**Figure 5A**). The inhibition of Ccr9 signaling resulted in a significant decrease of T cells in the inflamed gut, 56.7 v/s 22.7, to an amount similar to that observed in control fish, 31.7 v/s 22.7, (**Figure 5B**). Likewise, the number of APC-T cell interactions observed in each gut section of fish treated with the inhibitor decreased compared to the inflammatory condition, 3.6 v/s 1.4 (p<0.001), and as observed for the number of T cells, the number of interactions was comparable to that detected in the control condition, 2.3 v/s 1.4 (p>0.05) (**Figure 5C**). Strikingly, the analysis of the distance from APC-T cell interaction to the muscularis mucosa revealed that the few APC-T cell interactions established in the treated gut, did it in the same preferred area as in control and inflamed gut (**Figure 5D**).

## Discussion

In mammals, secondary lymphoid organs present in the intestinal mucosa play a key role to fine-tune immune response to the different types of antigens they encounter in order to maintain homeostasis or induce an inflammatory response when corresponding. In teleost fish, intestinal mucosa is devoid of these structures, therefore the interaction between immune cells would occur randomly, anywhere in the lamina propria, which would be less efficiently than the process in mammals.

Here, we demonstrated that the presence of both harmless and inflammatory diet antigens in the intestine of the teleost fish medaka, trigger signals that promote the interaction of APCs and T cells in a specific area in the lamina propria of the intestinal mucosa, where they presumably perform local antigen presentation. We also showed that the Ccl25-Ccr9 axis not only regulate T cells homing to the intestine but play a central role in defining the specific region in the mucosa where APCs and T cells will interact. Although the number of APC-T cell interactions decreased by blocking *ccr9* signaling, the interactions occurred in the same area of the lamina propria as under the inflammation and control conditions, demonstrating the existence of specific area for interaction. In zebrafish, using the same soybean meal-based diet, we detected an increase in intestinal T cells concomitant to the upregulation of the *ccl25* mRNA level (13). In mammals, the CCR9-CCL25 axis also recruits T cells to a specific region of the intestinal mucosa. In the case of CD8^+^ T cells, they are recruited specifically within the intestinal epithelium, but not in the lamina propria (41–43). Moreover, *in vivo*-primed effector CD8^+^ T cells showed regionalized differences in their localization in the small intestinal epithelium, with decreased CCR9-dependent positioning in the distal section, where CCL25 has a reduced expression (43). Our results not only provided the first functional and direct evidence in fish that the CCR9-CCL25 axis regulate T cells recruitment to the gut, but that this signaling cascade also participate in the compartmentalization of the gut mucosal immune response, positioning T cells recruitment to a defined location in the lamina propria. On the other hand, the issue that after treating fish with the Ccr9 inhibitor some T cells still being recruited to the gut and to the area of interaction with APC, suggest that another signaling could also be involved in both processes. Of course, we do not rule out that the signaling blockage exert by the inhibitor was not complete. It is interesting that although the conservation in the Ccl25-Ccr9 axis function, as well as the *ccr9a* expression by T cells, the medaka *ccl25* orthologues genes are expressed by intestinal mesenchymal cells. In mammals, gut *ccl25* mRNA is restricted to the villus epithelial region between crypt and tip, in addition to the follicle-associated epithelium of PP and endothelial cells (44, 45). In this sense, *ccl25a* is also expressed in the gut epithelium, but without difference between the base and the tip of the villus. Hence, our results give mesenchymal cells an unprecedented significance in the activation of the intestinal mucosal immune response of fish.

Although there is no report pointing to the existence of an organized mucosal immune response in the gastrointestinal tract of teleost fish, the existence of organized mucosal associated lymphoid tissue in the gills of several fish species, known as interbranchial lymphoid tissue (ILT), has been reported (14, 15, 46). It is quite intriguing that being both gills and intestine first-line organs for antigens encounter, thus highly immunogenic, in teleost fish organized mucosal lymphoid tissue only evolved in gills. In this regard, even though the defined area in the medaka lamina propria where APCs-T cells interaction occur does not appear to be structurally delimited, it shares ILT function in relation to T cells concentration. Recent finding corroborating the existence of Gill-Associated Lymphoid Tissue (GIALT) in zebrafish (46) point to a more complex function of this structure compared to what we observed in the intestine of medaka. The GIALT suffer pronounced structural changes after infection, including a rapid reduction in the number of T/NK cells, followed by a marked repopulation after several days. Despite we have not followed the dynamic of T cells recruitment to the intestine during inflammation but just analyzed in one time point, we observed an increase in T cells as well as in the number of APC-T cells interaction in the preferred area, process which could mimic the later event observed in ILT. On the other hand, it is important to consider that the immune response activation in ILT was triggered by a virus infection both in Atlantic salmon and zebrafish, process characterized by rapid an acute response. On the contrary, in medaka we induce intestinal inflammation by food intake, response that is slow and chronic. In light of the results obtained, it could be that because of the nature of the food-triggered inflamed process, the formation of lymphoid structures is not obvious or simply does not occur.

Another relevant aspect that could influence the detection of lymphoid structures is the developmental stage of the fish. An example of this is the epithelial anlage found next to the cloacal in Atlantic salmon, which resemble the bursa of Fabricius in birds. As such, after developing into a lymphoepithelial tissue, it subsequently regressed following sexual maturation (18). Thus, in adult fish will not be present. Our results indicated that embryonic developed lymphoid structures were not present in the medaka gut, but there is still the possibility that postembryonic structures, such as cryptopatches (CP), could be formed after proper stimulus. In mice, CP develop postnatally in a process influenced by the presence of dietary antigens and microbiota (47). They consist of small cluster of CCR6^+^ ILC3 and CD11c^+^CX3CR1^+^ myeloid cells, and only very few B cells, T cells, and stromal cell networks (48, 49). Thus, despite in our experimental strategy fish were exposed to food antigens for a couple of days and the gut started to be colonized by the microbiota, therefore at least some CP should be formed, the transgenic lines used to analyze APC-T cell interaction may not label all of them, in particular B cells are not analyzed in this study. In general, and due to gut immune cells just started to be characterized in fish, neither the different subpopulations that exist nor the marker genes that identify them are known in detail, therefore we cannot rule out that some APCs or T cells are not labeled with the transgenic lines used.

In conclusion, although the absence of structured lymphoid aggregates in the medaka intestine, the mucosal immune response induced by the presence of dietary antigens, in particular the interaction between APCs and T cells, did not take place randomly in the lamina propria but occurred in a defined region.

## Data Availability Statement

The original contributions presented in the study are included in the article/**Supplementary Material**. Further inquiries can be directed to the corresponding authors.

## Ethics Statement

The animal study was reviewed and approved by Animal Bioethics Committee of the Universidad Andres Bello, Chile (certificate number: 020/2016).

## Author Contributions

NA, JB, BB, CF, and JW contributed to the design of the experiments. NA, RA, JB, KL, and CF performed the experiments. BB, CF, and JW provided reagents and oversaw the work. All authors interpreted the data. NA and CF co-wrote the manuscript. All authors read and edited the manuscript. All authors contributed to the article and approved the submitted version.

## Funding

This work was supported by fellowships from the Alexander von Humboldt Foundation (to CF) and Heidelberg Graduate School for Life Sciences (to KL). The laboratory of BB is supported by the German Research Foundation (BA 5766/3-1) and that from CF by the FONDECYT Grant 1210903.

## Conflict of Interest

The authors declare that the research was conducted in the absence of any commercial or financial relationships that could be construed as a potential conflict of interest.

## Publisher’s Note

All claims expressed in this article are solely those of the authors and do not necessarily represent those of their affiliated organizations, or those of the publisher, the editors and the reviewers. Any product that may be evaluated in this article, or claim that may be made by its manufacturer, is not guaranteed or endorsed by the publisher.

## Supplementary Material

Supplementary Movie 1 Time-lapse *in vivo* imaging of the posterior intestine in the double Tg(il-12p40b:mTq2; lck:GFP) showing T cells (green) and APC (cyan) behavior during inflammation.
