# Supplementary material for: Antigen-Presenting Cells and T Cells Interact in a Specific Area of the Intestinal Mucosa Defined by the Ccl25-Ccr9 Axis in Medaka
Source: Front Immunol. 2022 Feb 3;13:812899. doi: 10.3389/fimmu.2022.812899 (PMC8853713; doi:10.3389/fimmu.2022.812899)
Supplement: Supplementary file 2 [file DataSheet_1.pdf]

Supplementary Material

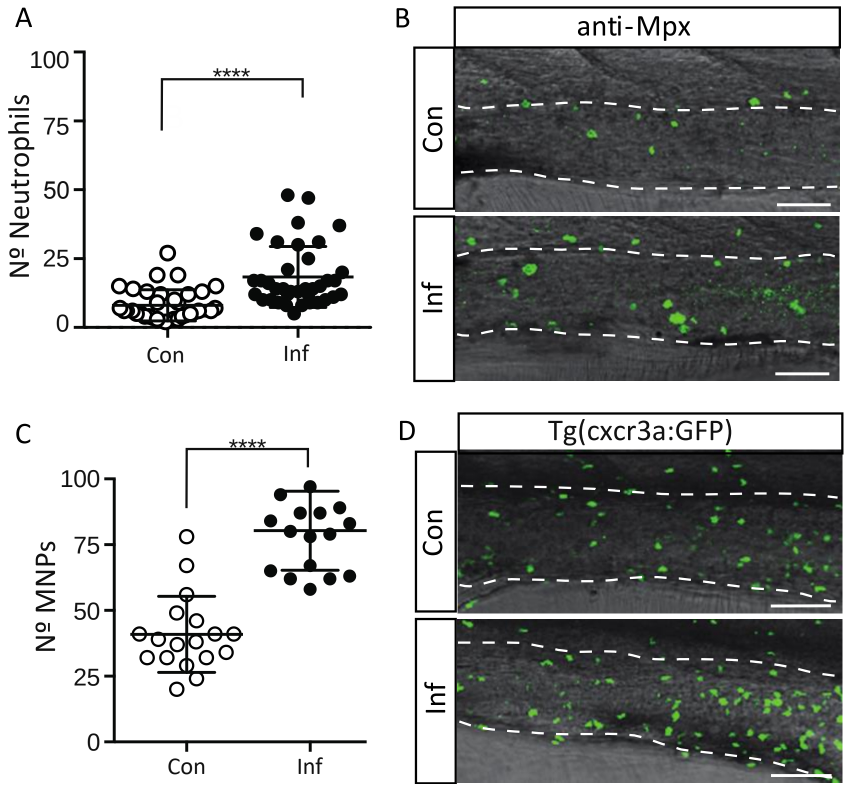

Supplementary Figure 1. Increased number of myeloid cells in the posterior gut in response to inflammation. (a) Quantification of the number of neutrophils in the posterior intestine in the control (Con) and inflammatory (Inf) condition, revealed by anti Mpx (Myeloperoxidase) immunofluorescence. Each dot represents a fish. (b) Representative images of the posterior intestine in control and inflammatory conditions showing anti Mpx immunofluorescence result. (c) Quantification of the number of mononuclear phagocytes (MNP) in the posterior intestine in the control (Con) and inflammatory (Inf) condition, revealed by anti GFP immunofluorescence on Tg(cxcr3a:GFP) fish. Each dot represents a fish. (d) Representative images of the posterior intestine in control and inflammatory conditions showing anti GFP immunofluorescence result. Each dot represents an individual animal. Statistical analysis was performed with the Mann-Whitney test. ****$p<0.0001$. Scale bars 50µm. All assays were performed at least three independent times.

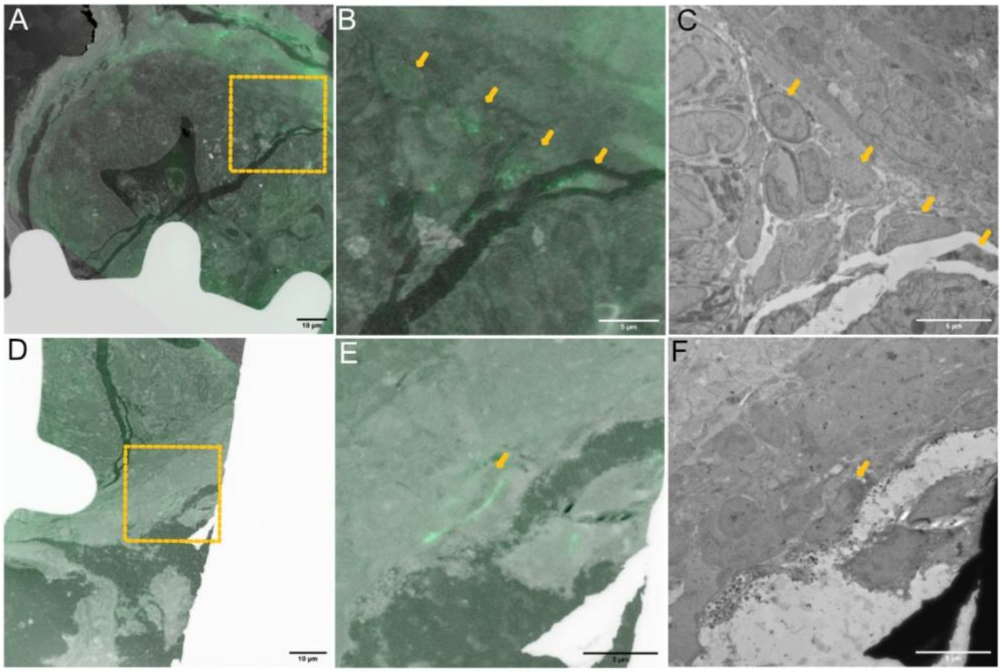

Supplementary Figure 2. Location of Ccl25b+ cells in the gut mucosa. Correlative light and electron microscopy (CLEM) of intestine from Tg(ccl25b:GFP), 20 days post hatch. Both widefield fluorescence microscopy (FM) and electron microscopy (EM) was performed on HM20 embedded, 80 nanometer thick sections placed on finders grids to aid in the correlation. A and D) Overview images of cross sections through the posterior intestine. Overlay of FM and EM data. EM data contrast inverted to visualize FM signal. Dashed rectangle indicates area magnified in B and E. B) Arrows indicate GFP positive cells adjacent to the lamina propria. C) EM data of the same area as B at higher magnification. Arrows indicate the same cells as in B. E) Arrow indicate GFP positive cells within the lamina propria. F) EM data of the same area as E at higher magnification. Arrow indicate the same cell as in E. Scale bars in A and D 10 µm; in B, C, E, F 5 µm.

Supplementary Table 1: Sequence of the primers used for quantitative PCR in this study

**Gene**	**Forward primer (5'-3')**	**Reverse primer (5'-3')**
ef1a	AAGTTCGAGAAGGAAGCCGC	TGATGGTCACATAGTACTTGC
ccl25a	ACATCTCTGCCCTCGTCTTC	TTGGCATCAAGGTTCTCCTTC
ccl25b	TCACACCCTCGTCTTGCTTC	ACACAACAGCTCTGATGTTGC
ccr9a	CATCAACTCCACTTCATCTCC	TGTCATTGTCCTCGTAATCATC
il12b	TGGAGGAAACTACACTTGTCAC	CTTCCAGGTGCAGTGAAAGGAGC
mhciib	GAGCGCCAGGAAAGCTCAGAAGG	GTAGAAGTCGTAGACGCTGCAGAC
tcrb	ACGAGGTACGGCACATCCTA	AACTTGTTGTCTGAGGTCCACTC
